# The impact of aortic root rotation on the position of the fibrous trigones on the mitral annulus

**DOI:** 10.1093/icvts/ivaf047

**Published:** 2025-02-27

**Authors:** Atsushi Sugaya, Shingo Hirao, Go Yamashita, Jiro Sakai, Tatsuhiko Komiya

**Affiliations:** Department of Cardiovascular Surgery, Kurashiki Central Hospital, Kurashiki, Japan; Department of Cardiovascular Surgery, Kurashiki Central Hospital, Kurashiki, Japan; Department of Cardiovascular Surgery, Kurashiki Central Hospital, Kurashiki, Japan; Department of Cardiovascular Surgery, Kurashiki Central Hospital, Kurashiki, Japan; Department of Cardiovascular Surgery, Kurashiki Central Hospital, Kurashiki, Japan

**Keywords:** aortic root rotation, mitral valve surgery, mitral annulus, atrioventricular conduction disturbance, computed tomography

## Abstract

**Objectives:**

Aortic root (AoR) rotation is a significant factor influencing the structures around the aortic valve and the atrioventricular conduction system. However, its relationship with the mitral valve remains unexplored. The goal of this study was to investigate the impact of AoR rotation on the mitral annulus, particularly the fibrous trigones, and on the atrioventricular conduction system following mitral valve surgery.

**Methods:**

We retrospectively reviewed 100 patients who underwent mitral valve surgery in which electrocardiography-gated computed tomography angiography scans were used to identify rotational variants of the AoR. AoR rotation was classified as clockwise, central or counterclockwise relative to the atrial septum. The positions of the fibrous trigones and postoperative atrioventricular conduction disturbances were analysed.

**Results:**

The distance from the right fibrous trigone to the right edge of the mitral annulus was shortest in the clockwise group and longest in the counterclockwise group (clockwise vs central vs counterclockwise: 6.1 ± 2.0 mm vs 7.4 ± 1.9 mm vs 8.7 ± 1.5 mm, *P *<* *0.001). The incidence of new-onset atrioventricular and bundle branch blocks was significantly higher in patients with AoR rotation (clockwise vs central vs counterclockwise: 63.2% vs 4.1% vs 21.9%, *P *<* *0.001).

**Conclusions:**

AoR rotation influences the position of the fibrous trigone and is a risk factor for postoperative atrioventricular conduction disturbances. Computed tomography is a valuable tool for assessing AoR rotation and mitral annulus morphology.

## Graphical Abstract

**Figure:**
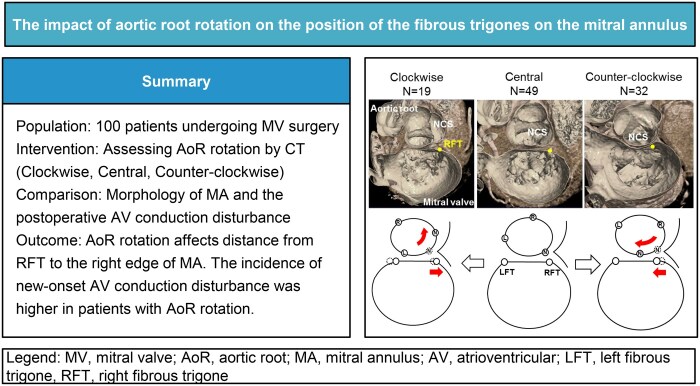

## INTRODUCTION

The prevalence of mitral regurgitation (MR) is reported to be globally high among valvular heart diseases [1]. Mitral valve repair and replacement are crucial in the treatment of mitral valve disease, offering excellent outcomes [2, 3]. However, the sutures used in these procedures can damage the bundle of His near the mitral annulus and the right fibrous trigone (RFT), potentially leading to postoperative atrioventricular conduction disturbances [4]. Additionally, serious complications such as detachment of the prosthetic valve or ring and paravalvular leakage can occur, underscoring the critical importance of precise mitral annulus capture during mitral valve operations [5, 6].

Recent reports suggest that aortic root (AoR) rotation can be identified through computed tomography (CT) and may pose a risk for right atrial perforation and tricuspid valve injury during an AoR operation [7, 8]. In cases with AoR rotation, caution is exercised when implanting a transcatheter aortic valve due to its impact on the anatomy of the atrioventricular conduction system and the morphological changes it induces in the membranous septum [9, 10]. Advancements in CT imaging technology have further facilitated comprehensive assessments of the mitral valve annulus, in addition to the aortic valve [11]. Although the aortic and mitral valve annuli are closely related components of the heart’s fibrous skeleton, the impact of AoR rotation on mitral valve annulus morphology remains unclear. Furthermore, the influence of anatomical alterations in the conduction system resulting from AoR rotation on atrioventricular conduction disturbances following mitral valve surgery has also not been established [12].

The objective of this retrospective study was to elucidate the relationship between AoR rotation and the mitral annulus, with a particular focus on the positions of the left and RFTs. This analysis was conducted using electrocardiography (ECG)-gated multislice CT scan images. Furthermore, the goal of the study was to determine the incidence of conduction system failure following mitral valve surgery.

## MATERIALS AND METHODS

### Ethics statement

The study adhered to the principles outlined in the Declaration of Helsinki and its subsequent amendments. This retrospective observational study was conducted at a single centre and was approved by the institutional review board of the Kurashiki Central Hospital (Approval No. 4453) on 6 August 2024. The requirement for informed consent was waived due to the retrospective nature of the study.

### Study population

**Figure 1:**
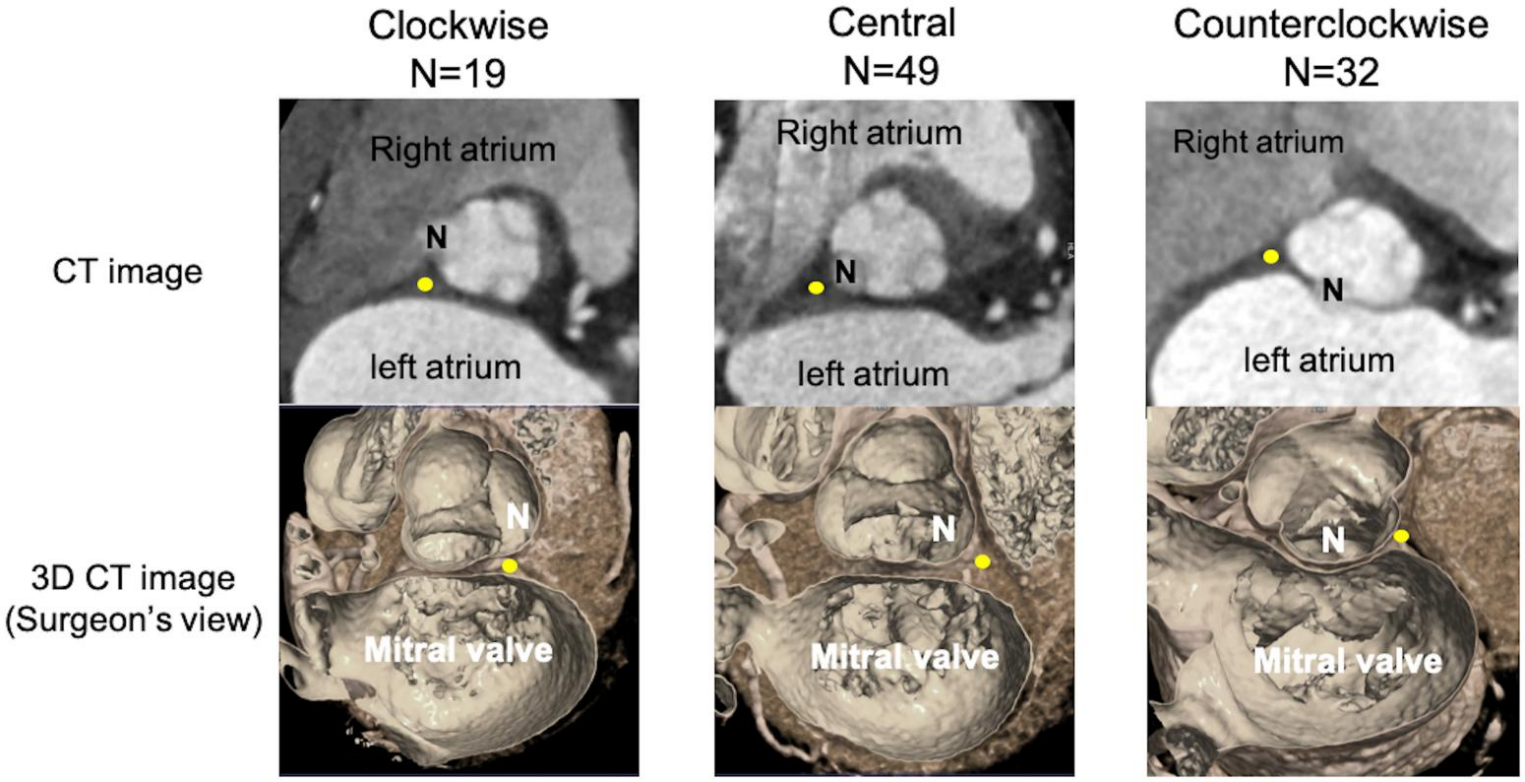
Aortic root rotation classification. Aortic root rotation is classified as clockwise, central or counterclockwise depending on the nadir position of the non-coronary sinus relative to the atrial septum (yellow dots). CT: computed tomography; N: non-coronary sinus; 3D: three- dimensional.

We analysed the AoR and the mitral valves in 182 patients with tricuspid aortic valves who underwent mitral valve surgery for MR at our facility between 2019 and 2023, using ECG-gated CT angiograms for assessment. Of these, 66 cases with inappropriate CT images (mostly caused by arrhythmias) and 16 cases with a history of prior mitral or aortic valve surgery were excluded. Baseline patient characteristics and echocardiographic data were obtained by reviewing medical records. AoR rotation was classified as clockwise, central or counterclockwise, based on the nadir position of the non-coronary sinus (NCS) relative to the atrial septum at the plane of the virtual basal ring, as observed in the CT images. It is important to note that the direction of AoR rotation is reversed when the aortic valve is observed intraoperatively compared to the CT assessment. Counterclockwise rotation was defined as a leftward rotation of the AoR relative to the atrial septum, whereas clockwise rotation was defined as a rightward rotation relative the atrial septum. Of the 100 patients, 49 had centrally positioned AoRs, 32 exhibited counterclockwise rotation and 19 exhibited clockwise rotation (Fig. 1).

### Computed tomography analysis

The data sets were acquired using ECG-gated contrast-enhanced CT angiographic scans (SOMATOM Definition Flash, Siemens, Munich, Germany). All CT analyses were performed on a commercially available workstation (Syngo Via, Siemens).

**Figure 2:**
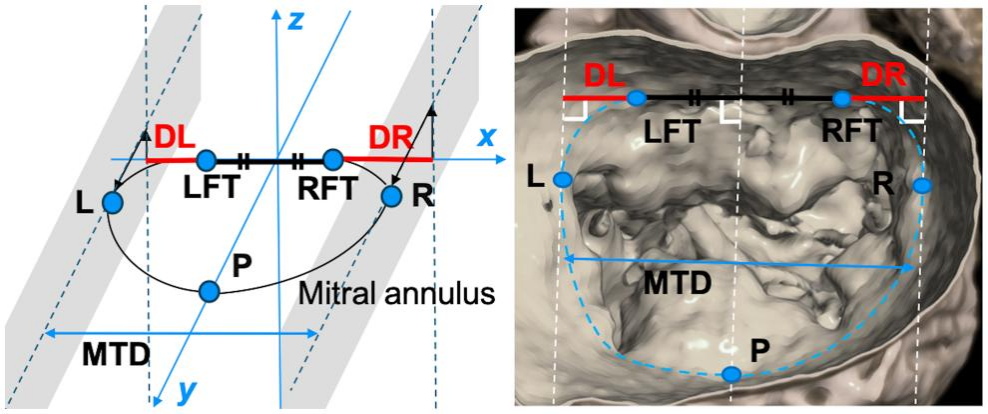
Mitral annulus analysed using three-dimensional computed tomographic reconstructions. The definition of the three-dimensional Cartesian coordinates in this study is as follows: The origin is the midpoint of the left fibrous trigone and the right fibrous trigone. The *XY* plane is the plane passing through the left and right fibrous trigones and point P on the mitral annulus. Point P is the intersection of the plane bisecting the left and right fibrous trigones perpendicularly and the mitral annulus. In the preceding coordinate system, the distance from the left fibrous trigone to the plane parallel to the *YZ* plane passing through the leftmost mitral annulus and the distance from the right fibrous trigone to the plane parallel to the *YZ* plane passing through the rightmost mitral annulus were measured. The blue dotted line in the right panel indicates the mitral annulus. DL: distance from the left fibrous trigone to the plane parallel to the YZ plane passing through the leftmost mitral annulus that had the smallest X-value among all points of the mitral annulus; DR: distance from the right fibrous trigone to the plane parallel to the YZ plane passing through the rightmost mitral annulus that had the largest X-value among all points of the mitral annulus; L: leftmost mitral annulus; LFT: left fibrous trigone; MTD: mitral transverse diameter; P: point on the mitral annulus; R: rightmost mitral annulus; RFT: right fibrous trigone.

The mitral annulus was evaluated using three-dimensional CT reconstruction. The three-dimensional Cartesian coordinates used in this study were defined as follows: The origin was the midpoint between the left fibrous trigone (LFT) and the RFT, respectively. The *XY* plane passed through the LFT, the RFT and a point P on the mitral annulus. Point P was defined as the intersection of the plane bisecting the LFT and RFT perpendicularly and the mitral annulus. In this coordinate system, the following distances were measured: DL, the distance from the LFT to the plane parallel to the *YZ* plane passing through the leftmost point of the mitral annulus, which had the smallest *X*-value among all points of the mitral annulus, and DR, the distance from the RFT to the plane parallel to the *YZ* plane passing through the rightmost point of the mitral annulus that had the largest *X*-value among all points of the mitral annulus (Fig. 2). Diastolic images were primarily selected for analysis to capture the mitral annulus in its potentially flattened state. The fibrous trigones were defined as the nadirs of the saddle-shaped annulus [13].

### Statistical analyses

All statistical analyses were performed using SPSS Statistics 20.0 software package for Windows (IBM Corp., Armonk, NY, USA). Continuous variables were expressed as mean ± standard deviation or median (interquartile range), depending on the distribution of the data. The Shapiro-Wilk test was used to assess normality. For the comparison of continuous variables among the 3 groups, one-way analysis of variance was used for normally distributed data, and the Kruskal-Wallis test was applied for non-normally distributed data. Categorical variables are presented as counts and percentages. Comparisons were performed using the *χ*^2^ test or the Fisher exact test (for frequencies <5). Univariate logistic regression analysis was conducted to identify the risk factors for new-onset transient atrioventricular conduction disturbances following a mitral valve operation. No prior power or sample size calculations were performed for this study. Post hoc power analyses, using a two-group *t*-test with a bilateral significance level of *P *<* *0.05, revealed the following: mean DR and DR/mitral transverse diameter (MTD) were 65.7% and 81.1% (clockwise vs central), 92.3% and 89.6% (central vs counterclockwise) and 99.7% and 99.8% (clockwise vs counterclockwise). Similarly, the power to detect differences in the mean DL and DL/MTD was 60.9% and 66.3% (clockwise vs central), 14.0% and 6.4% (central vs counterclockwise) and 28.5% and 53.7% (clockwise vs counterclockwise).

## RESULTS

### Baseline characteristics

Preoperative patient characteristics are summarized in Table 1. Patients in the clockwise group were generally older and exhibited a higher prevalence of hypertension compared to those in the other groups. However, no statistically significant differences were observed in other baseline clinical variables. ECG data showed no significant differences among the 3 groups in terms of basic conduction parameters and the incidence of atrial fibrillation, atrioventricular block (AVB) or bundle branch block (BBB). Echocardiographic findings revealed no significant differences in the dimensions of the left atrium and left ventricle, left ventricular function or the presence of valvular disease across the 3 groups.

**Table 1:** Preoperative patient characteristics

	Clockwise, *N* = 19	Central, *N* = 49	Counterclockwise, *N* = 32	*P*-value
Clinicals				
Age (years)	72.0 (71.0–79.0)	65.0 (55.0–74.0)	62.0 (57.0–70.0)	0.006
Female	8 (42.1)	19 (38.8)	9 (28.1)	0.514
BMI (kg/m^2^)	23.0 (20.5–25.9)	22.9 (21.0–24.6)	23.5 (20.9–24.8)	0.982
Renal impairment (eGFR < 45)	3 (15.8)	6 (12.2)	6 (18.8)	0.708^a^
Haemodialysis	0 (0.0)	1 (2.0)	2 (6.2)	0.583^a^
Diabetes	3 (15.8)	12 (24.5)	8 (25)	0.778^a^
Hypertension	16 (84.2)	33 (67.3)	15 (46.9)	0.021
Dyslipidaemia	10 (52.6)	19 (38.8)	10 (31.2)	0.318
COPD	9 (47.4)	22 (44.9)	15 (46.9)	0.976
NYHA functional classes III and IV	2 (10.5)	10 (20.4)	6 (18.9)	0.141^a^
Coronary vessel disease	3 (15.8)	9 (18.4)	3 (9.4)	0.569^a^
Pacemaker implant	0 (0.0)	2 (4.1)	0 (0.0)	0.683^a^
Active infective endocarditis	1 (5.3)	0 (0.0)	5 (15.6)	0.006^a^
Electrocardiogram data				
Heart rate (bpm)	71.0 (65.0–79.0)	70.5 (63.0–80.8)	73.0 (58.0–87.0)	0.837
PR interval (ms)	163.0 (149.0–188.0)	167.0 (150.0–180.3)	159.0 (143.0–193.0)	0.905
QRS interval (ms)	105.0 (97.0–114.0)	105.0 (97.0–110.5)	103.0 (93.5–109.5)	0.676
First-degree AVB	1 (5.3)	0 (0.0)	2 (6.2)	0.181^a^
Bundle branch block				0.198^a^
Complete left BBB	1 (5.3)	0 (0.0)	0 (0.0)	
Left anterior hemiblock	0 (0.0)	1 (2.0)	2 (6.2)	
Left posterior hemiblock	0 (0.0)	0 (0.0)	1 (3.1)	
Complete right BBB	2 (10.5)	4 (8.2)	1 (3.1)	
Incomplete right BBB	0 (0.0)	0 (0.0)	2 (6.2)	
Atrial fibrillation	2 (10.5)	5 (10.2)	0 (0.0)	0.130^a^
Echocardiography data				
Left atrial dimension (mm)	46.0 (42.0–50.0)	43.0 (40.5–47.0)	44.0 (38.3–48.5)	0.573
Left ventricular diastolic dimension (mm)	49.0 (44.0–53.0)	52.0 (47.0–57.0)	51.0 (47.3–56.8)	0.166
Left ventricular systolic dimension (mm)	29.0 (23.0–36.0)	30.0 (27.0–37.5)	32.5 (28.0–37.0)	0.171
Left ventricular ejection fraction (%)	65.0 (58.0–65.0)	67.0 (53.5–71.0)	63.0 (60.0–69.8)	0.624
Mitral valve regurgitation				0.742^a^
Mild	0 (0.0)	1 (2.0)	0 (0.0)	
Moderate	2 (10.5)	2 (4.1)	2 (6.2)	
Severe	17 (89.5)	46 (93.9)	30 (93.0)	

a Fisher exact test; values are presented as n (%) or median (IQR).

AVB: atrioventricular block; BBB: bundle branch block; BMI: body mass index; COPD: chronic obstructive pulmonary disease; eGFR: estimated glomerular filtration rate; IQR: interquartile range; NYHA: New York Heart Association.

The operative data are presented in Table 2. Among the patients, the superior trans-septal approach was used in only 1 patient in the counterclockwise group, whereas all other patients underwent the left atrial approach. Mitral valve repair was the predominant procedure across all 3 groups, and no significant differences were observed in the types or sizes of the prosthetic rings and valves used. Concomitant operations involving the aortic valve, the tricuspid valve and arrhythmia were performed as needed, with no statistically significant differences observed among the groups. Notably, there were no perioperative deaths.

**Table 2:** Operative data

	Clockwise, *N* = 19	Central, *N* = 49	Counterclockwise, *N* = 32	*P*-value
Emergency	0 (0.0)	1 (2.0)	1 (3.1)	0.743
CPB time (min)	203.0 (161.0–253.0)	201.0 (174.5–246.0)	202.0 (150.0–238.8)	0.766
Cross-clamp time (min)	141.0 (114.0–174.0)	133.0 (115.0–157.5)	130.0 (107.5–161.0)	0.711
Mini-thoracotomy	11 (57.9)	34 (69.4)	23 (71.9)	0.561
Approach				0.510^a^
Left atrial	19 (100.0)	49 (100.0)	31 (69.9)	
Superior trans-septal	0 (0.0)	0 (0.0)	1 (3.1)	
Mitral valve repair	18 (94.7)	47 (95.9)	29 (90.6)	0.746^a^
Ring size (mm)				0.051^a^
28	3 (16.7)	6 (12.8)	2 (6.9)	
30	6 (33.3)	21 (44.7)	3 (10.3)	
32	6 (33.3)	13 (27.7)	17 (58.6)	
34	2 (11.1)	5 (10.6)	5 (17.2)	
36	1 (5.5)	1 (2.1)	2 (6.9)	
38	0 (0.0)	1 (2.1)	0 (0.0)	
Ring type				0.235^a^
Full	2 (11.1)	10 (21.3)	2 (6.9)	
Partial	16 (88.9)	37 (78.7)	27 (93.1)	
Mitral valve replacement	1 (5.3)	2 (4.1)	3 (9.4)	0.746^a^
Valve size (mm)				>0.999^a^
27	0 (0.0)	0 (0.0)	1 (33.3)	
31	1 (100.0)	2 (100.0)	1 (33.3)	
33	0 (0.0)	0 (0.0)	1 (33.3)	
Valve type				0.600^a^
Mechanical	0 (0.0)	0 (0.0)	1 (50.0)	
Biological	1 (100.0)	2 (100.0)	1 (50.0)	
Aortic valve surgery	1 (5.3)	2 (4.1)	1 (3.1)	0.510^a^
Tricuspid valve surgery	8 (42.1)	10 (20.4)	6 (18.8)	0.141^a^
Maze surgery	3 (15.8)	8 (16.3)	3 (9.4)	0.741^a^
Coronary artery bypass grafting	2 (10.5)	7 (14.3)	3 (9.4)	0.918^a^

a Fisher exact test. Values are presented as *n* (%) or median (IQR).

CPB: cardiopulmonary bypass; IQR: interquartile range.

### Computed tomography examination of the mitral valve

**Figure 3:**
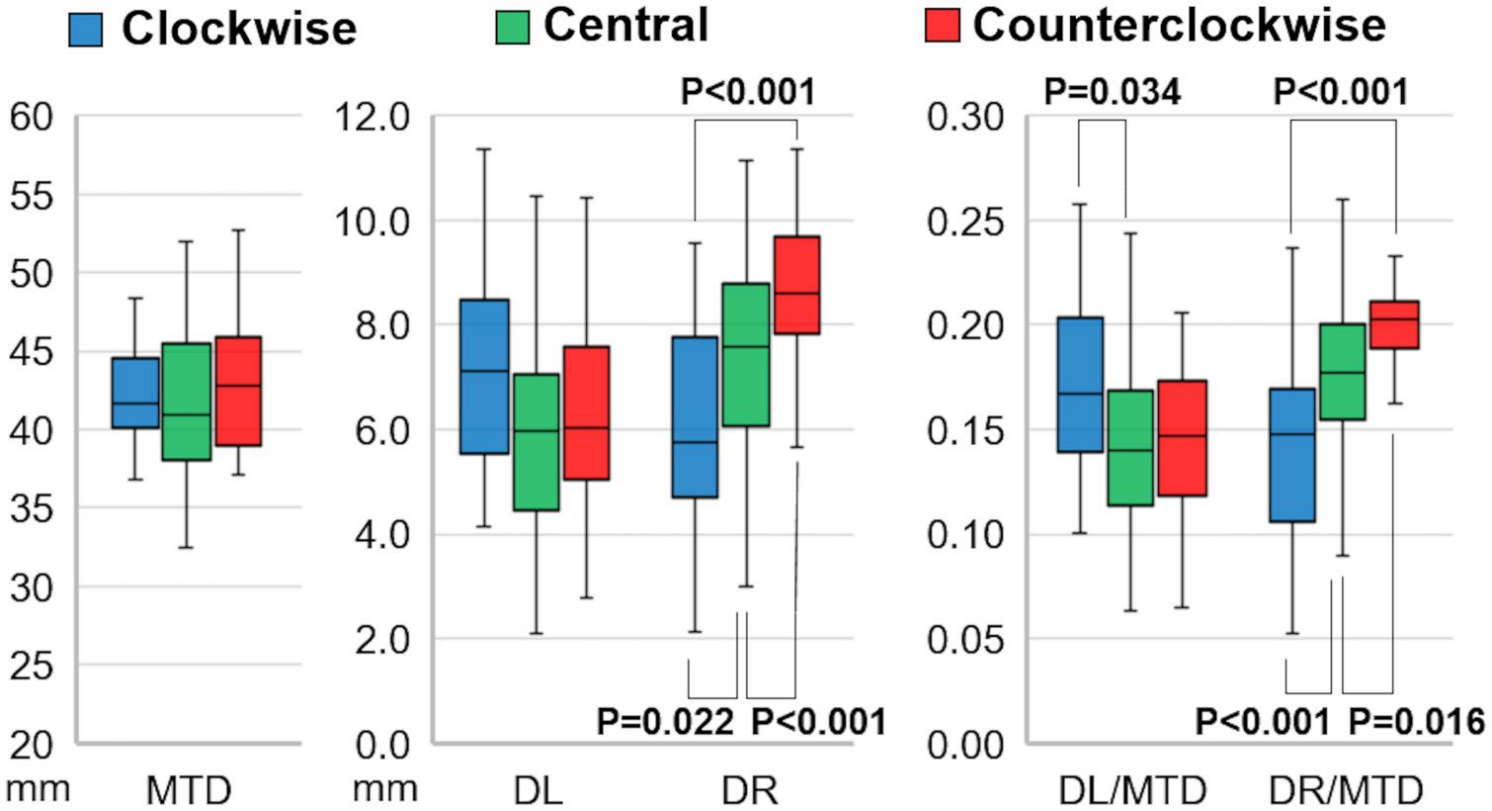
Distances from the fibrous trigones to the edges of the mitral annulus. DL: the distance from the left fibrous trigone to the plane parallel to the YZ plane passing through the leftmost edge of the mitral annulus; DR: the distance from the right fibrous trigone to the plane parallel to the YZ plane passing through the rightmost edge of the mitral annulus; MTD: mitral transverse diameter.

MTD showed no statistically significant differences among the 3 groups (clockwise vs central vs counterclockwise: 42.3 ± 3.4 mm vs 41.5 ± 4.9 mm vs 43.3 ± 4.7 mm, *P *=* *0.210). DR was the smallest in the clockwise group and the largest in the counterclockwise group (clockwise vs central vs counterclockwise: 6.1 ± 2.0 mm vs 7.4 ± 1.9 mm vs 8.7 ± 1.5 mm, *P *<* *0.001) (Fig. 3). A similar result was observed in the ratio of DR to MTD (DR/MTD), with the clockwise group showing the smallest value and the counterclockwise group showing the largest value (clockwise vs central vs counterclockwise: 0.144 ± 0.045 vs 0.178 ± 0.036 vs 0.199 ± 0.022, *P *<* *0.001). There were no significant differences in DL among the 3 groups (clockwise vs central vs counterclockwise: 7.1 ± 1.9 mm vs 5.9 ± 2.0 mm vs 6.3 ± 2.0 mm, *P *=* *0.096). However, the ratio of DL to MTD (DL/MTD) was largest in the clockwise group (clockwise vs central vs counterclockwise: 0.168 ± 0.041 vs 0.141 ± 0.040 vs 0.144 ± 0.036, *P *=* *0.039; Fig. 3).

### Incidence of atrioventricular conduction disturbance

The incidence of new-onset transient BBB was significantly higher in the clockwise group (clockwise vs central vs counterclockwise: 31.6% vs 2.0% vs 9.4%, *P *<* *0.001). Similarly, the occurrence of new-onset transient second-degree or third-degree AVB was more frequent in both the clockwise and counterclockwise groups (clockwise vs central vs counterclockwise: 42.1% vs 2.0% vs 12.5%, *P *<* *0.001). Detailed data on BBB and AVB are provided in Supplementary Table S1, and comparable findings were observed in cases of isolated mitral valve operations (Supplementary Table S2). Univariate logistic analysis identified counterclockwise rotation and age as significant risk factors for new-onset transient BBB and AVB following mitral valve surgery. Additionally, concomitant tricuspid valve surgery was associated with an increased incidence of postoperative atrioventricular conduction disturbances (Table 3). No patients required postoperative pacemaker implants for these conduction disturbances.

**Table 3:** Risk factors for new-onset transient atrioventricular conduction disturbance

	Branch bundle block: 10/100 (10%) univariate analysis		2- or 3-Degree atrioventricular block: 13/100 (13%) univariate analysis	
Variable	Odds ratio (95% CI)	*P*-value	Odds ratio (95% CI)	*P*-value
Baseline characteristics, ECG data and echocardiography				
Age (years)	1.08 (1.00–1.16)	0.050	1.07 (1.01–1.14)	0.031
Female sex	0.74 (0.18–3.06)	0.678	0.49 (0.13–1.91)	0.306
Renal impairment (eGFR < 45)	0.60 (0.07–5.14)	0.644	3.07 (0.81–11.7)	0.100
Diabetes	0.82 (0.16–4.17)	0.812	0.57 (0.12–2.79)	0.571
Hypertension	2.43 (0.49–12.1)	0.279	0.89 (0.27–2.94)	0.843
Active infective endocarditis	1.89 (0.20–18.0)	0.580	3.77 (0.62–23.1)	0.151
Heart rate (bpm)	1.00 (0.95– 1.04)	0.831	1.02 (0.98–1.05)	0.381
Preoperative BBB	1.63 (0.31–8.58)	0.568	2.07 (0.49–8.72)	0.320
Preoperative AVB	Unconverged^a^		Unconverged^a^	
Left atrial diameter (mm)	1.06 (0.98–1.15)	0.148	0.99 (0.91–1.07)	0.779
Left ventricular ejection fraction (%)	1.01 (0.95–1.08)	0.717	1.02 (0.97–1.09)	0.441
Aortic root rotation				
Clockwise rotation (versus central)	22.2 (2.45–200.7)	0.006	34.9 (3.95–308.7)	0.001
Counterclockwise rotation (versus central)	4.97 (0.49–50.0)	0.174	6.86 (0.73–64.4)	0.092
Operative procedure				
Cross-clamp time	1.01 (0.99–1.02)	0.361	1.00 (0.99–1.02)	0.910
Mitral valve replacement	1.89 (0.20–18.0)	0.580	Unconverged^a^	
Full ring (vs partial ring)^b^	1.74 (0.32–9.38)^b^	0.520	1.05 (0.21–5.32)^b^	0.957
Aortic valve surgery	3.22 (0.30–34.3)	0.332	Unconverged^a^	
Tricuspid valve surgery	3.74 (0.98–14.3)	0.054	3.29 (0.98–11.0)	0.054
Maze surgery	1.63 (0.31–8.58)	0.568	1.14 (0.22–5.78)	0.877

a Calculation was not feasible because there were no events.

b This group, including mitral valve repair; *n* = 94.

AVB: atrioventricular block; BBB: bundle branch block; CI: confidence interval; ECG: electrocardiography; eGFR: estimated glomerular filtration rate.

## DISCUSSION

**Figure 4:**
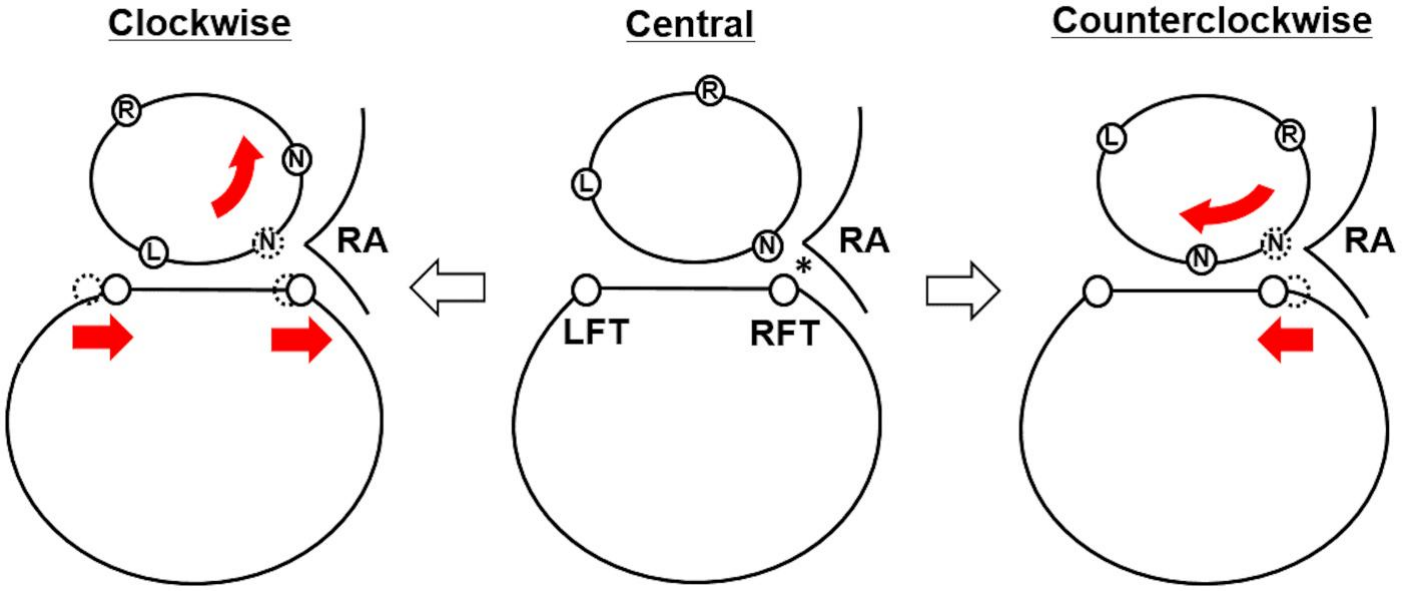
The differences in the distances between the fibrous trigones and the mitral annulus across different aortic root rotation groups. In the central group, the right fibrous trigone exhibited a tendency to have a longer distance from the right edge of the mitral annulus than from the left. This phenomenon was more pronounced in cases of counterclockwise rotation. In contrast, the distance of the left fibrous trigone from the left edge of the mitral annulus was longer in the clockwise rotation group compared to the central group. * atrial septum; L: nadir position of the left aortic sinus; LFT: left fibrous trigones; N: nadir position of the non-aortic sinus; R: nadir position of the right aortic sinus; RA: right atrium; RFT: right fibrous trigones.

Variations in the rotation of the AoR were identified through analysis of ECG-gated contrast-enhanced CT angiographic scans. The observed frequency of AoR rotation was 42% central, 19% clockwise and 39% counterclockwise. These findings highlight the utility of CT imaging in precise characterization of AoR rotation and its relationship with mitral annulus morphology. In the central group, the RFT tended to be at a greater distance from the right edge of the mitral annulus compared to the left edge. Furthermore, this asymmetry was more pronounced in patients with a counterclockwise rotation. In contrast, in the clockwise group, the distance of the LFT from the left edge of the mitral annulus was longer compared to that in the central group (Fig. 4). Clinical implications were evident in the association between AoR rotation and postoperative atrioventricular conduction disturbances. Patients with AoR rotation—both clockwise and counterclockwise—exhibited a higher incidence of transient atrioventricular conduction disturbances following mitral valve surgery compared to those with centrally aligned AoR.

Tretter *et al.* [10] analysed aortic valve rotation in 26 cadaveric normal hearts and reported frequencies of 54% normal, 31% counterclockwise and 15% clockwise, consistent with the findings of the present study. In contrast, Oishi *et al.* [8], in a report of cases with an enlarged AoR, reported 40.4% clockwise, 5.8% counterclockwise and 56% normal rotation, whereas Miazza *et al.* [7] observed 3.3% clockwise, 13.3% counterclockwise and 83.3% normal. These discrepancies may have stemmed from the differences in the primary diseases or the use of commissures as a reference point for classifying AoR rotation in the previous studies. Handa *et al.* [14], analysing AoR rotation in patients with MR using transoesophageal ECG, reported clockwise rotation in 2.4%, central in 83.3% and counterclockwise rotation in 14.3%. This study differs from the present study in that they relied for the definition of AoR on the position of the commissure between the left coronary sinus and the NCS relative to the centre of the anterior mitral leaflet and on the use of echocardiography. Based on the relationship between the AoR and the mitral anterior leaflet, they were classified as lateral appearance and centre appearance. In the cases of lateral appearance, AoR rotation was not assessed. Lateral appearance is possibly classified as a counterclockwise rotation according to the definition of AoR rotation in the present study. Therefore, the frequency of counterclockwise rotation was expected to be similar to that of the present study. The present study highlights the importance of the relationship between the AoR, the cardiac fibrous skeleton and the conduction system. The nadir of the NCS was specifically utilized as a marker to improve the anatomical and clinical understanding of AoR rotation.

The effect of AoR rotation on the location of the fibrous trigone, as observed in this study, is a crucial factor in mitral valve annuloplasty. Given that ring dehiscence following mitral valve repair is less prevalent in the region of the fibrous component within the mitral annulus [5], it is essential to ensure that valve ring sutures are securely anchored to the fibrous trigone. From this perspective, preoperative assessment of AoR rotation could be valuable in achieving this goal. Moreover, when using a partial ring in mitral valve repair, careful consideration of the ring form relative to the position of the individual RFT is essential. Further long-term follow-up studies are needed to determine whether selecting prosthetic rings tailored to the mitral annulus shape can effectively prevent remote ring dehiscence.

It is imperative to consider the impact of tricuspid, aortic and arrhythmic operations on the atrioventricular conduction system. However, this study highlights that the clockwise rotation poses a higher risk for postoperative atrioventricular conduction disturbances compared to other operative procedures. Notably, no cases required postoperative pacemaker implants, and AVB resolved spontaneously in many patients. This result indicates that the disturbances were not caused by direct procedural damage to the conduction system. It has been hypothesized that the anatomical proximity of the mitral annulus to the atrioventricular conduction system, which facilitates the propagation of inflammatory changes associated with prosthetic valve or ring implantation, contributes to the high incidence of disturbances in patients with AoR rotation. Additionally, the inherent fragility of the stimulatory conduction system may also play a role in this phenomenon [9].

### Study limitations

This study has several limitations. First, it was a retrospective study conducted at a single centre with a limited number of cases. The relatively small sample size posed challenges in implementing advanced statistical methods, such as multivariate analysis, to account for potential confounding factors. Moreover, the post hoc power to detect the difference in mean DL and DL/MTD was low. Second, due to the lack of data on the long-term outcomes, further studies are required to assess the remote occurrence of related events. To address these limitations, it is necessary to prospectively investigate long-term outcomes with larger patient cohorts and sufficient statistical power of detection. Finally, the evaluation of the mitral annulus on CT was highly sensitive, leading to instances of suboptimal imaging. Further advancements in CT imaging technology are anticipated to improve the diagnostic accuracy and reliability in the future.

## CONCLUSION

In conclusion, AoR rotation was found to significantly influence the position of the fibrous trigones on the mitral annulus. AoR rotation is identified as a risk factor for postoperative atrioventricular conduction disturbances and BBBs in patients undergoing mitral valve operations.

## Supplementary Material

ivaf047_Supplementary_Data

## Data Availability

The data utilized in this study are included within the article. Additional data can be provided by the corresponding author upon reasonable request.

## Notes

Presented at the 38th Annual Meeting of the European Association for Cardio-Thoracic Surgery, Lisbon, Portugal, October 9–12, 2024.

## ABBREVIATIONS

AoR: Aortic root
AVB: Atrioventricular block
BBB: Bundle branch block
CT: Computed tomography
DL: The distance from the LFT to the plane parallel to the YZ plane passing through the leftmost mitral annulus
DR: The distance from the RFT to the plane parallel to the YZ plane passing through the rightmost mitral annulus
ECG: Electrocardiography
LFT: Left fibrous trigone
MR: Mitral regurgitation
MTD: Mitral transverse diameter
NCS: Non-coronary sinus
RFT: Right fibrous trigone

## References

[1] Iung B , Delgado V, Rosenhek R et al; EORP VHD II Investigators. Contemporary presentation and management of valvular heart disease: the EURObservational Research Programme Valvular Heart Disease II Survey. Circulation 2019;140:1156–69. 31510787 10.1161/CIRCULATIONAHA.119.041080

[2] Uchino G , Murakami H, Mukohara N et al Modes of the bioprosthetic valve failure of the porcine and pericardial valves in the mitral position. Eur J Cardiothorac Surg 2022;62:ezab506. 34875043 10.1093/ejcts/ezab506

[3] Lazam S , Vanoverschelde J-L, Tribouilloy C et al; MIDA (Mitral Regurgitation International Database) Investigators. Twenty-year outcome after mitral repair versus replacement for severe degenerative mitral regurgitation: analysis of a Large, Prospective, Multicenter, International Registry. Circulation 2017;135:410–22. 27899396 10.1161/CIRCULATIONAHA.116.023340

[4] Carpentier A , Adams DH, Filsoufi F. Carpentier’s Reconstructive Valve Surgery. Amsterdam, The Netherlands: Elsevier, 2010,339–41.

[5] Noack T , Kiefer P, Vivell N et al Annuloplasty ring dehiscence after mitral valve repair: incidence, localization and reoperation. Eur J Cardiothorac Surg 2020;57:300–7. 31369069 10.1093/ejcts/ezz219

[6] Genoni M , Franzen D, Vogt P et al Paravalvular leakage after mitral valve replacement: improved long-term survival with aggressive surgery? Eur J Cardiothorac Surg 2000;17:14–9. 10735406 10.1016/s1010-7940(99)00358-9

[7] Miazza J , Winkel D, Thieringer F et al Aortic root rotation: morphological analysis of the aortic root with three-dimensional computed tomography. Eur J Cardiothorac Surg 2024;65:ezae040. 38310332 10.1093/ejcts/ezae040 PMC10931524

[8] Oishi K , Arai H, Oi K et al The rotational position of the aortic valve: implications for valve-sparing aortic root replacement. Eur J Cardiothorac Surg 2022;62:ezac179. 35293582 10.1093/ejcts/ezac179

[9] Tretter JT , Burbano-Vera NH, Najm HK. Multi-modality imaging evaluation and pre-surgical planning for aortic valve-sparing operations in patients with aortic root aneurysm. Ann Cardiothorac Surg 2023;12:295–317. 37554720 10.21037/acs-2023-avs2-0040 PMC10405341

[10] Tretter JT , Mori S, Saremi F et al Variations in rotation of the aortic root and membranous septum with implications for transcatheter valve implantation. Heart 2018;104:999–1005. 29146623 10.1136/heartjnl-2017-312390

[11] Blanke P , Naoum C, Webb J et al Multimodality imaging in the context of transcatheter mitral valve replacement: establishing consensus among modalities and disciplines. JACC Cardiovasc Imaging 2015;8:1191–208. 26481845 10.1016/j.jcmg.2015.08.004

[12] Anderson RH , Cook AC, Spicer DE, Hlavacek AM, Backer CL, Tretter JT. Wilcox’s Surgical Anatomy of the Heart. Cambridge, UK: Cambridge University Press, 2024.

[13] Blanke P , Dvir D, Cheung A et al Mitral annular evaluation with CT in the context of transcatheter mitral valve replacement. JACC Cardiovasc Imaging 2015;8:612–5. 25937198 10.1016/j.jcmg.2014.07.028

[14] Handa K , Kawamura M, Yoshioka D et al Impact of the aortomitral positional anatomy on atrioventricular conduction disorder following mitral valve surgery. J Am Heart Assoc 2024;13:e035826. 39158546 10.1161/JAHA.124.035826 PMC11963948

