# Supplementary material for: The impact of aortic root rotation on the position of the fibrous trigones on the mitral annulus
Source: Interdiscip Cardiovasc Thorac Surg. 2025 Feb 27;40(3):ivaf047. doi: 10.1093/icvts/ivaf047 (PMC11951097; doi:10.1093/icvts/ivaf047)
Supplement: ivaf047_Supplementary_Data [file ivaf047_supplementary_data.zip › CORRECT_Supplementary_material.docx]

**Supplementary Table S1: New-onset transient atrioventricular conduction disturbance**

|  | Clockwise  N = 19 | Central  N = 49 | Counterclockwise  N = 32 | P value |
| --- | --- | --- | --- | --- |
| New-onset BBB |  |  |  | 0.001* |
| Complete left BBB | 0 (0.0) | 1 (2.0) | 0 (0.0) |  |
| Incomplete left BBB | 2 (10.5) | 0 (0.0) | 1 (3.1) |  |
| Complete right BBB | 2 (10.5) | 0 (0.0) | 0 (0.0) |  |
| Incomplete right BBB | 2 (10.5) | 0 (0.0) | 2 (6.2) |  |
| None | 13 (68.5) | 48 (98.0) | 29 (90.6) |  |
| New-onset AVB |  |  |  | < 0.001* |
| Second-degree AVB | 1 (5.3) | 0 (0.0) | 3 (3.1) |  |
| Third-degree AVB | 7 (36.8) | 1 (2.0) | 1 (9.4) |  |
| None | 11 (57.9) | 48 (98.0) | 1. (87.5) |  |

*Fisher exact test; Values are presented as n (%); BBB, bundle branch block; AVB, atrioventricular block.

**Supplementary table S2: New-onset transient atrioventricular conduction disturbance in patients who underwent isolated mitral valve surgery**

|  | Clockwise  N=9 | Central  N=26 | Counterclockwise  N=21 | P value |
| --- | --- | --- | --- | --- |
| New-onset BBB |  |  |  | 0.010* |
| Incomplete left BBB | 1 (11.1) | 0 (0.0) | 1 (4.8) |  |
| Incomplete right BBB | 2 (22.2) | 0 (0.0) | 1 (4.8) |  |
| None | 6 (66.6) | 26 (100.0) | 19 (90.5) |  |
| New-onset AVB* |  |  |  | 0.002* |
| Second-degree AVB | 1 (11.1) | 0 (0.0) | 2 (9.5) |  |
| Third-degree AVB | 3 (33.3) | 0 (0.0) | 1 (4.8) |  |
| None | 5 (56.6) | 26 (100.0) | 18 (85.7) |  |

*Fisher exact test; Values are presented as n (%); BBB, bundle branch block; AVB, atrioventricular block.
